# Imaging characteristics of young age breast cancer (YABC) focusing on pathologic correlation and disease recurrence

**DOI:** 10.1038/s41598-021-99600-6

**Published:** 2021-10-12

**Authors:** Jeongmin Lee, Sung Hun Kim, Bong Joo Kang, Ahwon Lee, Woo-Chan Park, Jinwoo Hwang

**Affiliations:** 1 Department of Radiology, Seoul St. Mary’s Hospital, College of Medicine, The Catholic University of Korea, 222 Banpo-daero, Seocho-gu, Seoul, 06591 Republic of Korea; 2 Department of Hospital Pathology, Seoul St. Mary’s Hospital, College of Medicine, The Catholic University of Korea, Seoul, Republic of Korea; 3 Division of Breast-Thyroid Surgery, Department of Surgery, Seoul St. Mary’s Hospital, College of Medicine, The Catholic University of Korea, Seoul, Republic of Korea; 4 Philips Healthcare Korea, Seoul, Republic of Korea

**Keywords:** Breast cancer, Cancer imaging

## Abstract

The purpose of this study is to investigate imaging characteristics of young age breast cancer (YABC) focusing on correlation with pathologic factors and association with disease recurrence. From January 2017 to December 2019, patients under 40 years old who were diagnosed as breast cancer were enrolled in this study. Morphologic analysis of tumor and multiple quantitative parameters were obtained from pre-treatment dynamic contrast enhanced breast magnetic resonance imaging (DCE-MRI). Tumor-stroma ratio (TSR), microvessel density (MVD) and endothelial Notch 1 (EC Notch 1) were investigated for correlation with imaging parameters. In addition, recurrence associated factors were assessed using both clinico-pathologic factors and imaging parameters. A total of 53 patients were enrolled. Several imaging parameters derived from apparent diffusion coefficient (ADC) histogram showed negative correlation with TSR; and there was negative correlation between MVD and Ve in perfusion analysis. There were nine cases of recurrences with median interval of 16 months. Triple negative subtype and low CD34 MVD positivity in Notch 1 hotspots showed significant association with tumor recurrence. Texture parameters reflecting tumor sphericity and homogeneity were also associated with disease recurrence. In conclusion, several quantitative MRI parameters can be used as imaging biomarkers for tumor microenvironment and can predict disease recurrence in YABC.

## Introduction

Breast cancer has become one of the leading causes of death in Asian women and its incidence in Asia has been increasing steadily^1–4^. Along with this trend, the different epidemiology of Asian breast cancer is now being focused on. Especially, its peak age is one of the biggest differences with breast cancer of the West. Its peak age is in the 70 s in the West, whilst it is in the late 40 s in the East. Moreover, the proportion of premenopausal women is higher compared to the West. Despite the higher incidence in the West, the proportion of breast cancer in young women under 40 s and premenopausal women is higher in the East than in the West^5–8^.

These epidemiologic differences could affect the treatment of breast cancer. However, the treatment guidelines are mostly based on the characteristics of breast cancer of the West, which are focused on post-menopausal women. Recently, several clinical studies have been conducted to find proper treatment for Asian young age breast cancer (YABC) and some have shown significant results such as additional Ribociclib therapy to traditional endocrine therapy^9,10^. Treatments or screening guidelines based on these differences are needed for Asian women. For example, in Asian breast cancer, menopausal status should be considered in treatment and a more personalized consideration is needed for patients of childbearing age^5,6^.

Since young age is known as an independent risk factor for recurrence in breast cancer^11,12^, it is important to accurately evaluate the disease status when planning treatment for YABC. However, biopsy tissue often reflects only a portion of the tumor due to its heterogeneity. Imaging features or parameters on breast magnetic resonance imaging (MRI), unlike biopsy, are more accessible than pathologic characteristics and can noninvasively and comprehensively provide information of the entire tumor. However, there have been only a few studies on imaging characteristics of YABC.

Based on this background, we tried to assess how imaging parameters reflect pathologic features through MRI. Also, we investigated the imaging features associated with breast cancer recurrence in YABC.

## Result

### Patients

Sixty patients agreed and were enrolled in this study. Of the 60 patients, three withdrew their consent to participate in other clinical trials before MRI acquisition, and four withdrew their consent after MRI acquisition. Finally, 53 patients were enrolled. The median age of patients was 36 (range 23–39) years old and median period of follow up after surgery was 21.5 months (range 10–33). Of the finally enrolled 53 patients, 12 patients underwent neoadjuvant chemotherapy before surgery and 22 patients underwent adjuvant chemotherapy. Thirty-eight patients underwent breast conserving surgery followed by adjuvant radiation therapy. There were 51 cases of invasive ductal carcinoma and 2 cases of mucinous carcinoma. Based on pathologic report after surgery, stage 1A was the most common (n = 23) disease stage and luminal type was the most common tumor subtype by immunohistochemistry (n = 32). Eight patients underwent neoadjuvant chemotherapy and achieved complete remission before surgery. These patients were confirmed as stage 0 on pathology after surgery.

**Figure 1:**
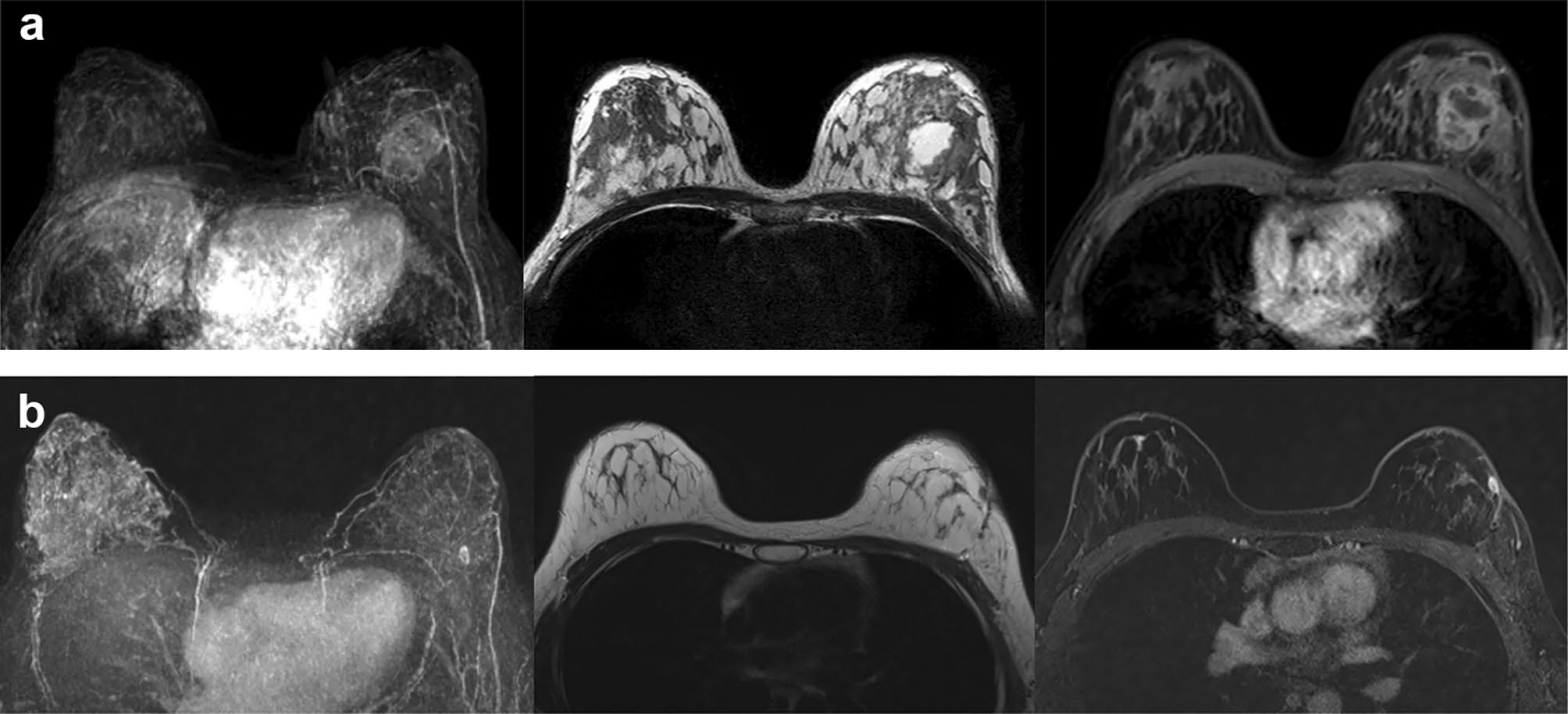
MRI of a 33-year-old female patient with left breast cancer (invasive ductal carcinoma, triple negative subtype). (**a**) Pre-treatment DCE-MRI. On MIP image, moderate BPE of both breasts and increased vascularity around the mass are noted. On non-fat saturated T2WI, mass shows internal necrotic change with underlying heterogenous fibroglandular tissue. On early phase of contrast enhanced fat saturated T1WI, mass shows oval circumscribed margin with internal heterogenous enhancement at 2 o’clock direction of left breast. (**b**) Post-operative DCE-MRI 12 months after surgery. On MIP image, a small circumscribed brightly enhancing nodule is noted at left breast. On non-fat saturated T2WI, an oval circumscribed nodule with low to intermediate signal intensity is noted near the previous excision site. This nodule shows rim enhancement pattern on early phase of contrast enhanced fat saturated T1WI. This lesion was confirmed as recurrent breast cancer (IDC, triple negative subtype) by US-guided core needle biopsy.

At a median follow up of 21.5 months, there were 9 cases of recurrence. Five cases recurred at ipsilateral breast and one case recurred at contralateral breast. One case recurred as an ipsilateral axillary metastasis and two cases recurred as distant metastasis. Apart from these nine recurrent cases, there was one case with endometrial cancer as a second primary cancer. Recurrent cases were identified within average of 13.8 months (median 16 months, range 1–31 months) after surgery. Among the nine recurrent cases, 5 were triple negative subtypes (Fig. 1). Other details of patient characteristics are described in Table 1.

**Table 1:** Clinico-pathologic characteristic of patients.

	Total (n = 53)	Non-recurrence group (n = 44)	Recurrence group (n = 9)	*p* value
Age (median)	36	36	35	
**Operation type**				1
Breast conserving surgery	38 (74.5)	31 (73.8)	7 (77.8)	
Mastectomy	13 (25.5)	11 (26.2)	2 (22.2)	
**BRCA mutation**				1
Negative	14 (63.6)	11 (64.7)	3 (60)	
Positive	8 (36.4)	6 (35.3)	2 (40)	
**Radiation therapy**				1
Yes	46 (88.5)	38 (88.4)	8 (88.9)	
No	6 (11.5)	5 (11.6)	1 (11.1)	
**Neoadjuvant chemotherapy**				0.185
Yes	12 (23.1)	8 (18.6)	4 (44.4)	
No	40 (76.9)	35 (81.4)	5 (55.6)	
**Adjuvant chemotherapy**				0.144
Yes	22 (42.3)	16 (37.2)	6 (66.7)	
No	30 (57.7)	27 (62.8)	3 (33.3)	
**Target therapy (Herceptin)**				0.33
Yes	9 (17.3)	9 (20.9)	0 (0)	
No	43 (82.7)	34 (79.1)	9 (100)	
**Adjuvant hormone therapy**				0.024
Yes	39 (76.5)	35 (83.3)	4 (44.4)	
No	12 (23.5)	7 (16.7)	5 (55.6)	
**Pathologic staging†**				0.366
Stage 0	4 (7.7)	4 (9.3)	0 (0)	
Stage I (1a, 1b)	24 (46.2)	21 (48.8)	3 (33.3)	
Stage II (2a, 2b)	22 (42.3)	16 (37.2)	6 (66.7)	
Stage III (3a)	2 (3.8)	2 (4.7)	0 (0)	
**Histologic type**				0.313
Invasive breast cancer, NST	51 (96.2)	43 (97.7)	8 (88.9)	
Invasive lobular carcinoma				
Mucinous carcinoma	2 (3.8)	1 (2.3)	1 (11.1)	
**Histologic grade††**				0.364
Grade 1	11 (20.8)	10 (22.7)	1 (11.1)	
Grade 2	18 (34)	16 (36.4)	2 (22.2)	
Grade 3	24 (45.3)	18 (40.9)	6 (66.7)	
**Estrogen receptor**				0.02
Positive	41 (77.4)	37 (84.1)	4 (44.4)	
Negative	12 (22.6)	7 (15.9)	5 (55.6)	
**Progesterone receptor**				0.044
Positive	39 (73.6)	35 (79.5)	4 (44.4)	
Negative	14 (26.4)	9 (20.5)	5 (55.6)	
**HER2**				0.175
Positive	8 (18.2)	8 (22.9)	0 (0)	
Negative	36 (81.8)	27 (77.1)	9 (100)	
Ki 67 (%)	41.9 ± 28.6	37 ± 25.6	57.6 ± 32.7	0.056
**Subtype**				0.006
Luminal	32 (61.5)	28 (65.1)	4 (44.4)	
HER2	10 (19.2)	10 (23.3)	0 (0)	
Triple negative	10 (19.2)	5 (11.6)	5 (55.6)	
**Tumor stromal ratio (TSR)**				0.645
Stroma predominant	9 (17.3)	7 (16.3)	2 (22.2)	
Tumor predominant	43 (82.7)	36 (83.7)	7 (77.8)	
**Stromal type**				0.579
Collagen	12 (24)	10 (24.4)	2 (22.2)	
Fibroblast	32 (64)	27 (65.9)	5 (55.6)	
Lymphocyte	6 (12)	4 (9.8)	2 (22.2)	
**Central necrosis**				0.456
Absent	47 (94)	39 (95.1)	8 (88.9)	
Present	3 (6)	2 (4.9)	1 (11.1)	
**Tumor infiltration lymphocyte (TIL)**				0.595
Low (< 50%)	43 (86)	36 (87.8)	7 (77.8)	
High (≥ 50%)	7 (14)	5 (12.2)	2 (22.2)	
Notch 1	9.7 ± 5.5	9.5 ± 5.3	11 ± 6.9	0.529
Notch1-DC	102.8 ± 28.9	105.7 ± 26.9	86.6 ± 36.4	0.042
EC Notch 1	0.1 ± 0.05	0.09 ± 0.05	0.12 ± 0.05	0.094
microvessel density (MVD)	114.1 ± 28.9	115.7 ± 28.6	105.2 ± 31.3	0.383
Notch tumor H score	16 ± 25.8	14.31 ± 25.1	25.7 ± 29.5	0.093

^†^Based on AJCC 7th, including yp stage.

^††^Nottingham histologic grade.

### Correlation between pathologic factors and imaging parameters

#### Apparent diffusion coefficient histogram and tumor-stroma ratio

Of the 52 patients who performed tumor-stroma ratio (TSR) analysis, 43 patients were stroma-poor type and 9 were stroma-rich type. Mean apparent diffusion coefficient (ADC) value of stroma-poor type was significantly lower than that of stroma-rich type (*p* = 0.006) (Table 2). Negative correlation was revealed among TSR and several parameters of ADC histogram: minimum value, mean value, median value, 5th percentile, 10th percentile, 25th percentile, 75th percentile and 90th percentile (Table 3).

**Table 2:** Mean ADC^†^ values according to pathologic parameters.

Pathologic parameters	Mean ADC values (SD) (× 10^−3^ mm^2^/s)	*p* value
**Tumor-stroma ratio**		
Stroma-poor (n = 43)	0.991 ± 0.053	0.006
Stroma-rich (n = 9)	1.391 ± 0.172	
**Stroma type**		
Collagenous (n = 12)	1.120 ± 0.143	0.561
Fibroblastic (n = 32)	1.009 ± 0.057	
Lymphocytic (n = 6)	1.144 ± 0.153	
**Central necrosis**		
Absent (n = 47)	1.061 ± 0.056	0.622
Present (n = 3)	0.946 ± 0.046	
**Tumor-lymphocytic infiltration 50%**		
Low (n = 43)	1.049 ± 0.058	0.79
High (n = 7)	1.087 ± 0.144	

^†^Apparent diffusion coefficient.

**Table 3:** Correlation coefficient between ADC^†^ values and tumor-stroma ratio.

ADC histogram	Correlation coefficient	*p* value
Minimum	− 0.4599	0.001
Maximum	− 0.1141	0.421
Mean	− 0.3467	0.018
Skewness	0.0935	0.021
Kurtosis	− 0.1153	0.51
5th percentile	− 0.3997	0.416
10th percentile	− 0.3916	0.003
25th percentile	− 0.3755	0.004
75th percentile	− 0.3159	0.006
90th percentile	− 0.2911	0.023
95th percentile	− 0.2694	0.036

^†^Apparent diffusion coefficient.

#### Perfusion parameters and endothelial notch 1 and microvessel density

The endothelial Notch 1 (EC notch 1) and microvessel density (MVD) analyses were performed in 45 patients of 53 patients. There was no association between perfusion parameters and EC notch 1. Of the multiple parameters from perfusion analysis, only the mean value of Ve showed negative correlation with MVD (correlation coefficient = −0.30, *p* < 0.046).

### Recurrence associated factors

#### Clinico-pathologic factors

There were 9 cases of tumor recurrence including loco-regional recurrence and distant metastases. The recurrence group showed significantly higher estrogen receptor (ER) negativity and progesterone (PR) negativity (*p* = 0.02, 0.044) than in non-recurrence group; and Ki-67 index tended to be higher in recurrence group (57.6 ± 32.7 [SD], *p* = 0.056). Lack of adjuvant hormone therapy was also higher in recurrence group (*p* = 0.024). The ER negativity, PR negativity and lack of adjuvant hormone therapy also showed significantly increased risk for disease recurrence (Table 4). Ten cases of the total 53 cases were triple negative subtypes. Of these 10 cases, 5 cases had disease recurrence accounting for 55.6% (5 cases of 9 recurrence cases) of all recurrences. In addition, triple negative subtype showed the highest risk of disease recurrence among clinical-pathological factors (OR 9.75, 95%CI 1.95–48.83], *p* = 0.019).

**Table 4:** Disease-recurrence associated clinico-pathologic factors.

Factors	Odds ratio (95% CI)	*p* value
Estrogen receptor negativity	6.61 (1.41–30.92)	0.016
Progesterone receptor negativity	4.86 (1.08–21.90)	0.039
Lack of adjuvant hormone therapy	6.25 (1.33–29.30)	0.020
Triple negative subtype	7.00 (1.38–35.83)	0.019
Notch1-CD34MVD (< 72.6)	9.00 (1.34–60.46)	0.020

**Figure 2:**
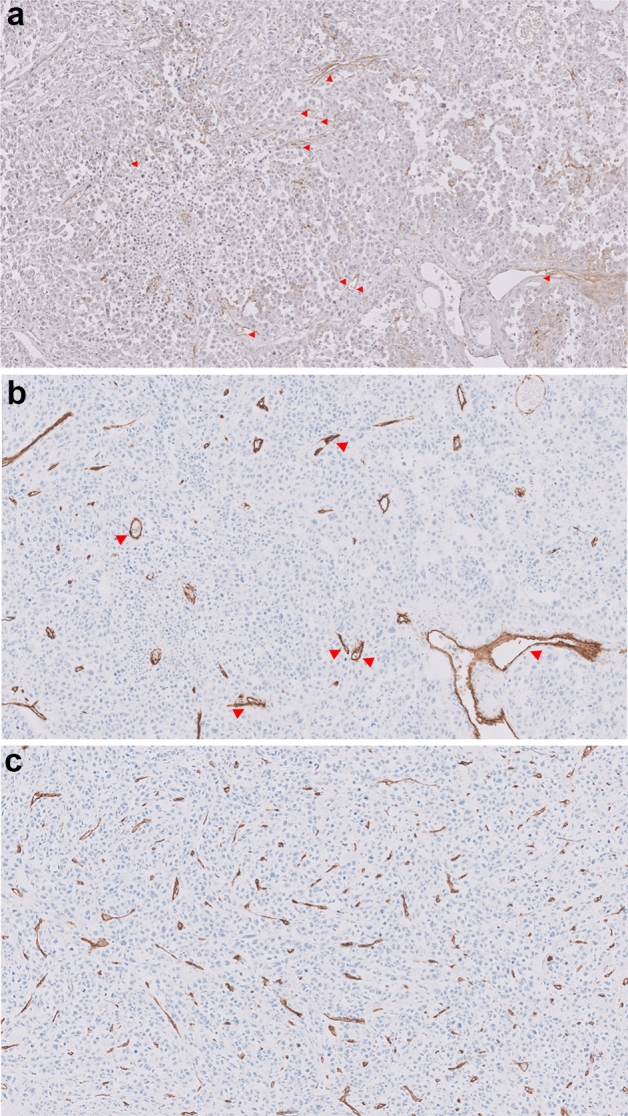
Low positive CD34 MVD in recurrent breast cancer. Immunohistochemical staining of Notch1 (**a**) and CD34 (**b**) at Notch 1 hotspots (original magnification ×200). Red arrow heads indicate Notch 1 positive microvessels (**a**), and some of CD34 positive microvessels are noted (**b**) among Notch 1 positive microvessels. In the same patient, (**c**) shows high level of CD34 MVD (brown colored) measurement in the entire tissue. However, the positivity of CD34 MVD in Notch 1 hotspots was as low as shown in (**b**). The patient was diagnosed as recurrent breast cancer 3 months after surgery.

In immunohistochemical staining, lower positivity of CD34 MVD in Notch1 hot spots was significantly associated with disease recurrence (OR 9.00, 95% CI 1.08–21.90, *p* = 0.02) (Fig. 2, Table 4).

#### Imaging factors

1. Qualitative parameters: morphologic characteristics

The mean size of the tumor was 2.9 cm (mean 2.88 cm ± 2.05 [SD]). In the recurrence group, the mean size of tumor was 3.9 cm (3.9 cm ± 1.7 [SD]), which was significantly larger than that of the tumor in non-recurrence group (mean 2.7 cm ± 2.1 [SD], *p* = 0.02). However, the increase in the mean size of tumors was not significantly associated with disease recurrence (*p* = 0.081).

More than half of the mass cases manifested as oval shape (n = 27, 57.5%) with circumscribed margin (n = 26, 55.3%). Except for one case of mucinous carcinoma which lacked enhancement, all mass lesions showed heterogenous or rim enhancement. However, these morphologic features showed no significant difference between recurrence and non-recurrence group.

The peritumoral edema was found only in 14 cases (26.4%). It was more frequently found in the recurrence group (55.6%, *p* = 0.044), and it was a significant factor for disease recurrence (OR 4.861, 95% CI 1.08–21.90, *p* = 0.039).

Mass shape, distribution of non-mass enhancement, number of prominent vessels around the index cancer, and other non-tumor factors showed no significant association with disease recurrence. The difference in whole qualitative parameters including morphologic characteristics of tumor and non-tumor factors between recurrence and non-recurrence groups is provided as Supplementary Table 1.

2. Quantitative parameters

From texture analysis, multiple parameters showed significant difference between recurrence and non-recurrence group (Supplementary Table 2). Among these parameters, low surface area to volume ratio calculated from T2-weighted imaging (T2WI) (OR 15.625, 95% CI 2.94–82.84, *p* = 0.001) and ADC map (OR 16.949, 95% CI 2.93–99.44, *p* = 0.002) showed the highest risk increase from other parameters. Of the first order features, only parameters calculated on the ADC map were correlated with disease recurrence; however, features such as entropy, kurtosis or skewness, which are commonly referred to describe tumor texture features, were not included. Of the second-order features, one gray level co-occurrence matrix (GLCM) feature calculated on the ADC map showed increased risk for the disease recurrence: inverse difference moment (IDM) (OR 5.625, 95% CI 1.23–25.76, *p* = 0.026), which is a measure of local homogeneity. In similar context, lower value of run length non uniformity normalized feature, a kind of homogeneity in image, showed the tendency to increase the risk of disease recurrence (OR 8.00, 95% CI 1.08–21.90, *p* = 0.059) (Table 5).

**Table 5:** Disease-recurrence associated imaging factors.

Factors	Odds ratio (95% CI)	*p* value
**Quantitative**		
Peritumoral edema positivity	4.86 (1.08–21.90)	0.039
**Qualitative**		
*Texture analysis: subtracted early phase of contrast enhanced fat saturated T1WI*^†^		
Original gray level run length matrix run length non uniformity normalized		
≥ 0.92	8.00 (0.92–69.45)	0.059
< 0.92	Reference	
*Texture analysis: T2WI*		
Original shape surface area to volume ratio		
< 0.388	15.60 (2.9378–82.8381)	0.001
≥ 0.388	Reference	
Original gray level run length matrix long run low gray level emphasis		
≥ 0.00616	0.104	0.040
< 0.00616	Reference	
*Texture analysis: ADC*^††^ *map*		
Original shape surface area to volume ratio		
< 0.31113	17.08 (2.9346–99.4468)	0.002
≥ 0.31113	Reference	
Original shape least axis		
≥ 17.429	6.000	0.023
< 17.429	Reference	
Original first order energy		
≥ 604,233,589	5.559	0.046
< 604,233,589	Reference	
Original first order total energy		
≥ 2,832,352,151	12.706	0.021
< 2,832,352,151	Reference	
Original first order 10th percentile		
≥ 851	4.861	0.039
< 851	Reference	
Original first order 90th percentile		
≥ 2243	7.778	0.010
< 2243	Reference	
Original first order maximum		
≥ 2128	12.706	0.021
< 2128	Reference	
Original first order mean		
≥ 1504.925	13.611	0.003
< 1504.925	Reference	
Original first order median		
≥ 1516	7.778	0.010
< 1516	Reference	
Original first order range		
≥ 2128	14.000	0.017
< 2128	Reference	
Original first order mean absolute deviation		
≥ 403.389	6.800	0.016
< 403.389	Reference	
Original first order root mean squared		
≥ 1653.109	7.778	0.010
< 1653.109	Reference	
Original first order SD		
≥ 352.99	6.767	0.027
< 352.99	Reference	
Original first order variance		
≥ 124,601.6	15.467	0.013
< 124,601.6	Reference	
Original gray level co-occurrence matrix inverse difference moment		
≥ 0.155	5.62 (1.23–25.76)	0.026
< 0.155	Reference	

^†^Weighted image.

^††^Apparent diffusion coefficient.

There was no significant association between perfusion parameters, and ADC histogram and recurrence in our study.

## Discussion

In this study, imaging characteristics of YABC were assessed focusing on their correlation with pathologic factors and on association with disease recurrence.

In the correlation between pathological factors and imaging parameters, TSR and ADC value, and MVD and Ve value showed correlation with each other. These were consistent with previous studies^13–15^. However, these pathologic factors and imaging parameters showed no significant correlation with disease recurrence in our study. The association between TSR and disease recurrence showed results that contradict the previous studies. In one previous study, stroma-rich type tumor showed higher relapse rate in triple negative breast cancer^16^. However, five cases of triple negative subtype with disease recurrence were all stroma-poor tumors in our study.

Although there was no significant association between EC Notch1 and image parameters, CD34 MVD in Notch1 hot spots showed negative correlation with disease recurrence. Despite that higher MVD showed association with poor prognosis of tumor in general, CD34 MVD showed contradictory result to previous studies^17^. This result may be due to the limitation of the small number of cases in our study. However, CD34 MVD in Notch 1 hot spots may be a new prognostic factor with different means from CD34 MVD measured in the entire tumor tissue. Further research is needed in the future for validation of this result.

According to previous studies on imaging characteristics of YABC, breast cancer under 40 years of age showed irregular shape with irregular or spiculated margin and internal heterogenous enhancement pattern on MRI^18,19^. These are typical findings of breast malignancy in average age women. However, more than half of the malignancies in our study manifested as mass lesions with oval shape with circumscribed margin, which is different from previous studies^20^. In addition, low value of surface area to volume ratio from T2WI and ADC map showed the highest correlation with recurrence on texture analysis. In other words, the more spherical the three-dimensional shape of the tumor is, the higher the association with recurrence. These morphologic features are similar to triple negative subtype breast cancer, which generally shows as mass with smooth or circumscribed margin^21,22^. Considering the high recurrence rate of triple negative subtype^23,24^, the results are consistent with previous studies. Moreover, triple negative subtypes accounted for more than half of the recurrence group in our study. Our results are not only consistent with previous studies, but also are more meaningful because they have been more objectively verified through texture analysis.

High value of IDM from ADC map also showed correlation with disease recurrence. This result is not consistent with the results of previous studies because the IDM indicates homogeneity of the tumor. In a previous study, the ADC value difference of tumor could reflect tumor heterogeneity; and high ADC value difference was associated with distant metastasis in breast cancer patients with mean age of 50 years old^25^. In this previous study, the ADC value difference was measured only on one section with the widest diameter of tumors. In our study, texture parameters of ADC map were measured in three dimensions to reflect the heterogeneity or homogeneity of the ADC value of the entire tumor. Another previous study has proven increased uniformity and decreased entropy in high histologic grade tumor^26^, though the texture parameters were measured on contrast-enhanced T1-weighted subtraction images.

These results, which are inconsistent with previous studies, may be the unique features of young age breast cancer because there are few studies on pathologic factors and quantitative MR parameters focusing on breast cancer in women under 40 years old.

Several texture parameters from ADC map showed significant correlation with disease recurrence whereas some previous studies showed that texture analysis of ADC map could not help predict the prognosis of breast cancer^27,28^. Diffusion weighted imaging (DWI) is emerging as a non-contrast imaging to replace contrast-enhanced MRI due to safety from gadolinium-based contrast agents. Thus, our results can be regarded as meaningful since many significant results were obtained from DWI. Based on this, DWI may have the potential to be a surveillance tool for high-risk young women who need annual contrast enhanced breast MRI by recommendation of American College of Radiology^29^.

There are few limitations in our study. Despite its prospective nature, this is a single center study; and the number of enrolled patients and event number are small, which could weaken the representatives of characteristics of YABC. In addition, the follow-up period after surgery was not enough, considering disease free survival of hormone receptor positive breast cancer. There were also some missing data in pathological factors and MR parameters.

Despite these limitations, this prospective study would have the novelty for description of imaging characteristics of YABC focusing on the correlation with pathologic factors and disease recurrence. Low CD34 MVD in Notch 1 hot spots and tumor homogeneity on ADC map, which showed correlation with disease recurrence, were contradictory to results of previous studies. These can be the unique features of YABC, although further validation would be needed with larger number of patients and longer follow-up period. In the situation where Asian breast cancer with a high proportion of young age women is rapidly increasing, studies to reveal the characteristics of YABC are necessary. Therefore, we expect this study to be the milestone of future studies that may reveal the characteristics of YABC.

## Materials and methods

### Patients

This prospective study was approved by institutional review board of Seoul St. Mary’s Hospital (reference number, KC16EISI0425) and assessments were carried out as per the rules of the Declaration of Helsinki of 1975, revised in 2013. From August 2017 to August 2019, patients diagnosed with invasive breast cancer under 40 years of age were the subjects for this study. Every patient was provided with informed consent before enrollment and agreed to undergo pre-treatment breast MRI with specific sequences in our institution for this study.

### MRI

MRIs were performed for patients in prone position using a 3-Tesla MR scanner (Ingenia, Philips Medical Systems, Best, The Netherlands) and a dedicated eight-channel phase-array coil. Images were obtained using the following sequences: (1) axial turbo spin-echo T2WI with TR/TE of 3624/80 ms, flip angle of 90°, field of view (FOV) of 320 × 320 mm^2^, matrix size of 532 × 306, slice thickness of 2 mm, and acquisition time of 2 min 23 s; (2) pre-contrast T1-weighted High Resolution Isotropic Volume Examination (THRIVE) with TR/TE of 9.0/2.0 ms, FOV of 300 × 300 mm^2^, matrix size of 256 × 204, slice thickness of 2 mm, flip angle of 5°, 10° and acquisition time of 2 min 45 s to determine tissue T1 relaxation time prior to the arrival of contrast agent; (3) dynamic contrast enhanced axial T1-weighted imaging (DCE-T1WI) with fat suppression with TR/TE of 9.0/2.0 ms, flip angle of 8°, slice thickness of 2.0 mm, and acquisition time of 5 min 30 s (temporal resolution 6 s) following an intravenous bolus injection of 0.1 mmol/kg gadobutol (Gadovist, Schering, Berlin, Germany) followed by a 20 ml saline flush; (4) delayed axial TRIVE with TR/TE of 4.5/2.0 ms, flip angle of 12°, slice thickness of 1.0 mm, FOV of 340 × 340 mm^2^, matrix size of 380 × 377 for evaluation of overall extent of tumor.

DWI was performed before dynamic contrast enhanced MRI, using a single-shot spin-echo EPI pulse sequence with TR/TE 12,043.5/102.3 ms, flip angle of 90°, FOV of 320 × 320 mm^2^, matrix size of 184 × 184, slice thickness of 3 mm, using three b values (b = 0, 1000 s/mm^2^). The ADC map was calculated with mono-exponential fit using a b value of 1000.

### Clinical and pathologic information

Medical records of all enrolled patients were reviewed. Clinical information included patient's age, date of surgery, the last date of outpatient clinic after surgery, date of disease recurrence, and the type of treatment received including surgery, chemotherapy, hormonal therapy and target therapy. Pathologic information of breast cancer included histologic type and grade, hormonal receptor status, human epidermal growth factor receptor 2 (HER2) status, Ki-67 index, and pathologic TNM staging by AJCC 7th.

Several additional pathologic analyses were performed including TSR, EC notch 1 and MVD of tumor. These factors were known as prognostic factors in previous studies^16,30,31^ and showed some correlation with imaging parameters: ADC values and perfusion parameters. EC Notch 1 has not been identified to correlate with imaging parameters to our knowledge. However, since this factor is already known as an angiogenesis-reflecting factor, we expect it to be able to predict the association with perfusion parameters; Ktrans, Kep and Ve.

These analyses were performed with surgical specimen. However, the tissue obtained from the core-needle biopsy at the time of cancer diagnosis was used if the patient underwent neoadjuvant chemotherapy. Staining of MVD and EC Notch 1 was omitted in cases of biopsy-tissue acquired in other hospitals.

Recurrence was defined as one of the followings: newly diagnosed breast cancer, axillary lymph node metastasis, or distant metastasis after surgery. Recurrent diseases were diagnosed by pathological confirmation when biopsy was possible; and in cases where biopsy was unavailable, other imaging modalities such as computed tomography (CT), or positron emission tomography (PET) -CT were used.

### Ethical approval

All procedures performed in studies involving human participants were in accordance with the ethical standards of the institutional and/or national research committee and with the 1975 Helsinki declaration and its later amendments or comparable ethical standards.

### Informed consent

Informed consent was obtained from all individual participants included in the study.

## Imaging analysis

### Morphologic analysis

Two breast expert radiologists prospectively analyzed morphologic characteristics of tumors on breast MRI in consensus, blind to the clinico-pathologic information of patients. The longest diameter of tumor, shape, margin, internal enhancement pattern, and kinetic curve were analyzed by BI-RADS lexicon on early and late dynamic phase of DCE-MRI. Presence of peritumoral edema and number of prominent vessels around the tumor, which were known as imaging prognostic factors by previous studies, were assessed on T2WI and on maximal intensity projection (MIP) image, respectively^32–34^. Fibroglandular tissue (FGT) pattern on T2WI, and background parenchymal enhancement (BPE) pattern on MIP images were also analyzed as non-tumor features in the contralateral breast, which was not involved by cancer.

### Quantitative analysis of MR parameters

#### Apparent diffusion coefficient histogram

**Figure 3:**
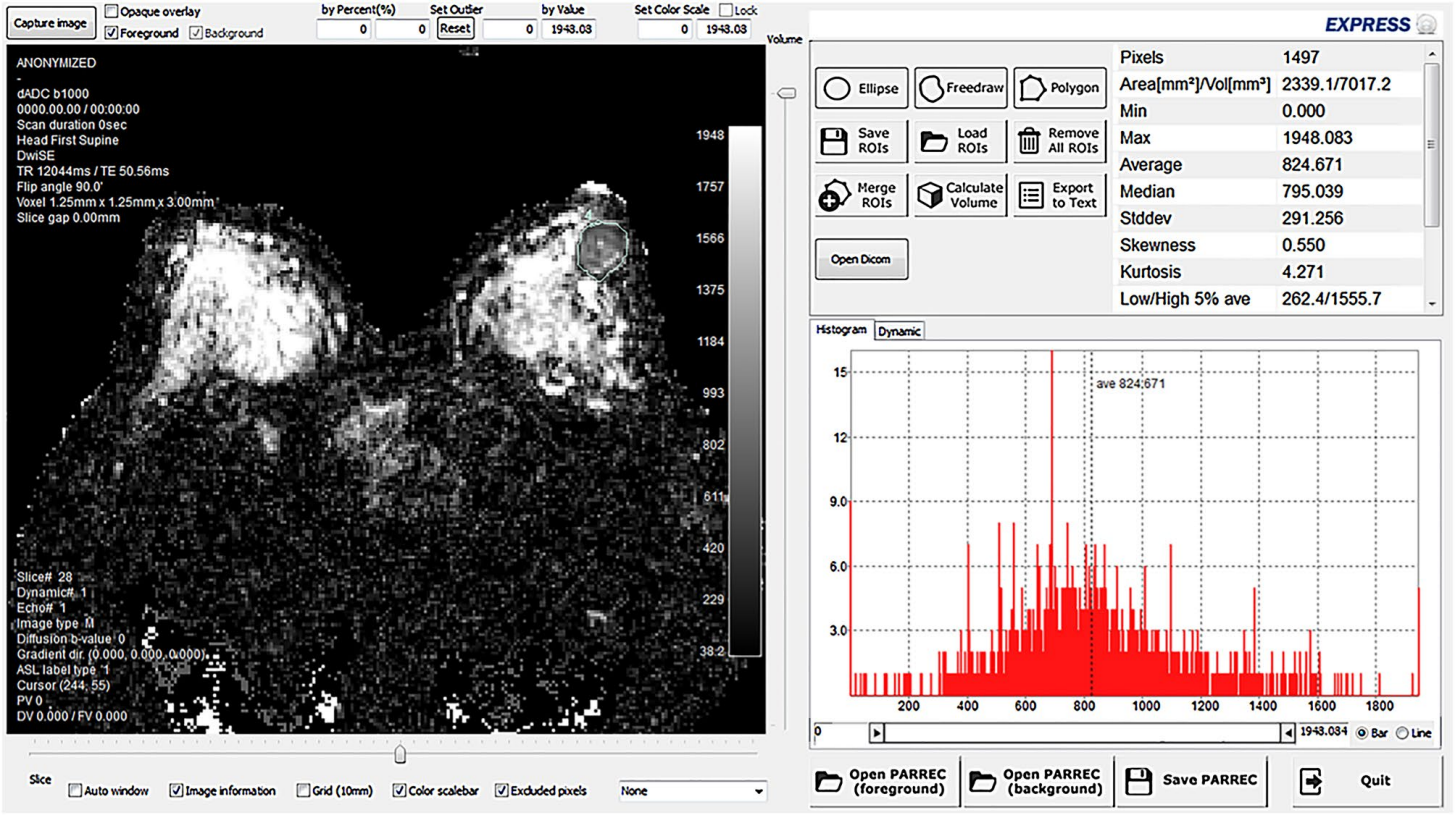
Analysis of ADC histogram of invasive breast cancer at subareolar region of left breast. The segmentation of tumor in ADC map was performed by manually drawing the ROIs in every single slice encompassing the entire tumor. The location of tumor was determined by referring to DWI and early dynamic phase images if the tumor location was confusing or not definite. After all ROIs were manually drawn, they were merged as VOI. Histogram analysis was performed on the VOI.

Post-processing was performed using In-house analysis software (EXPRESS; licensed by Jinwoo Hwang, Philips Healthcare Korea) to obtain ADC histogram of a defined tumor volume. Freehand type of region of interest (ROI) was manually drawn along the margin of the lesion for each image of ADC data. Then, a volume of interest (VOI) was defined as a stack of ROIs; and minimum, maximum, mean, 5th, 10th, 25th, 75th, 90th, 95th percentile ADC values, skewness and kurtosis of the pixels contained within the VOI were calculated (Fig. 3).

#### Perfusion parameter and texture analysis

The analysis for perfusion parameters was conducted in the same way as the previous study from our institution using a post-processing software (Olea Sphere, version 3.0, licensed by Olea Medical, La Ciotat, France)^20^.

**Figure 4:**
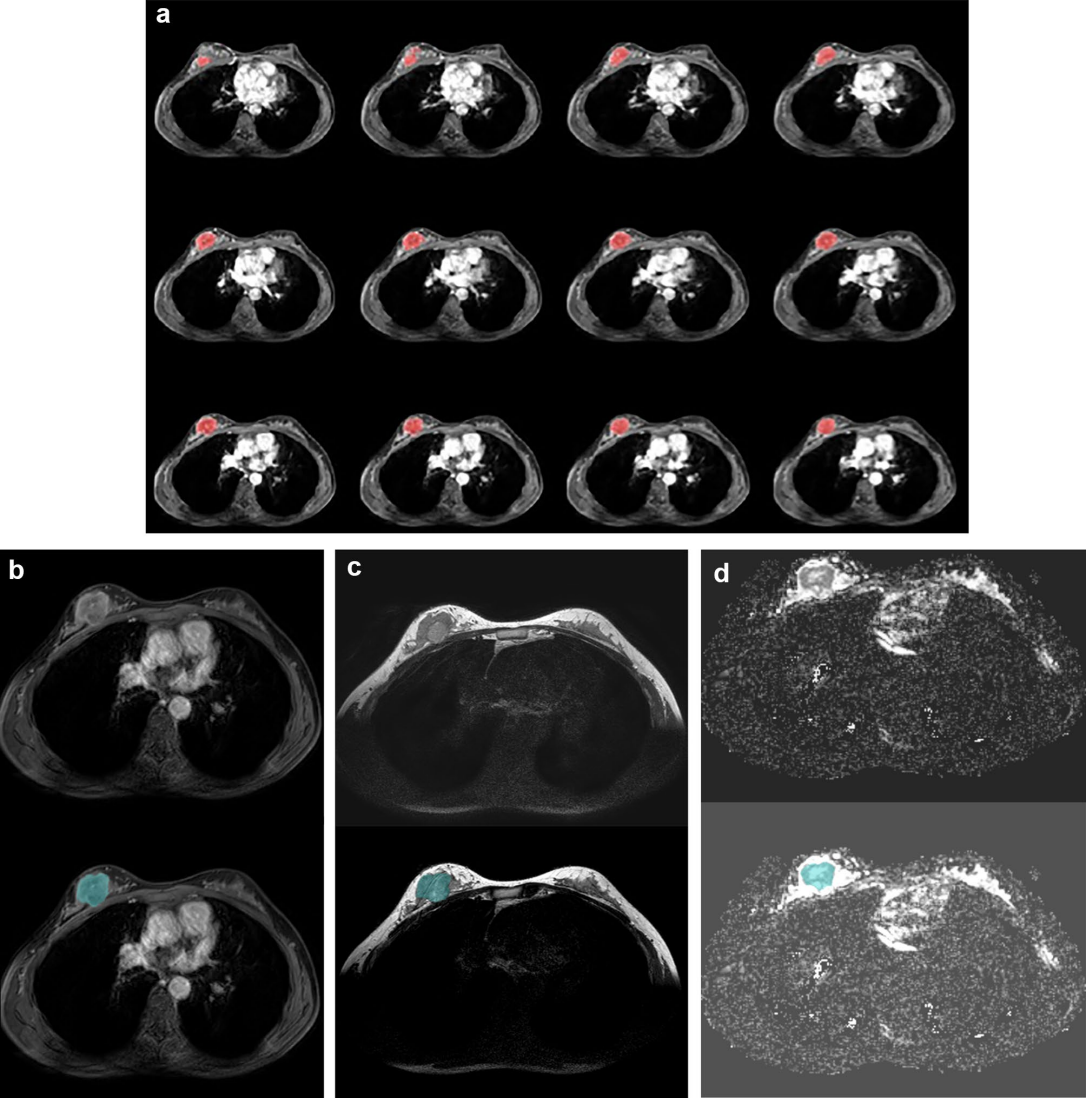
Texture analysis of invasive ductal carcinoma at 12 o’clock direction of right breast. Based on the VOI obtained from the perfusion parameters analysis (**a**), it was applied on subtracted early phase contrast enhanced T1WI (**b**), non-fat saturated T2WI (**c**), and ADC map (**d**). The color maps that do not match the tumor were manually modified using the original MR images of these sequences.

Texture analysis was also performed in Olea Sphere, with the same VOI drawn for perfusion parameter. We extracted first order features, gray level co-occurrence matrix (GLCM), and gray level run length matrix (GLRLM) from calculation on subtracted early dynamic phase T1WI, T2WI and ADC map. If the tumor was not clearly visualized on T2WI or ADC map, the VOI drawn on subtracted early dynamic phase T1WI was automatically applied at the similar location on T2WI or ADC map in the program and texture parameters were calculated (Fig. 4).

### Pathologic analysis and immunohistochemistry

#### Tumor-stroma ratio

One HE-stained slide of representative tumor was analyzed on microscopic examination. As previously determined, cases with stroma proportion of < 50% were considered as stroma poor and cases with stroma proportion of ≥ 50% were regarded as stroma rich (17).

#### Angiogenesis assessment with CD34 and Notch1 immunohistochemical staining

Four µm-thick sections for immunohistochemical staining of CD34 and Notch1 were obtained from formalin-fixed paraffin embedded tissue. The used primary antibodies were anti-CD34 mouse monoclonal antibody (clone QBEnd 10, 1:100 dilution, Dako, Glostrup, Denmark) and anti-Notch1 rabbit monoclonal antibody (clone D1E11, 1:50 dilution, CST, Danvers, USA). The CD34 staining was performed using the DAKO Omnis automated immunohistochemistry system (Dako). Tissue sections were deparaffinized with xylene, three times for 10 min, for Notch1 staining. Then, the sections were rehydrated using 100%, 95%, and 70% graded ethanol for 5 min, each after oven incubation at 60 °C for 1 h. Antigen retrieval was carried out in a pressure cooker (Electric Pressure Cooker CPC-600, Cuisinart, East Windsor, USA) for 20 min using 1× citrate buffer (pH 6.0). The endogenous peroxide activity was blocked by methanol-diluted 3% hydrogen peroxide for 15 min. Sections were incubated with the primary antibody for 1 h at room temperature (22–25 °C) in a humidified chamber. The immunoreaction signal was amplified and was revealed using the Polink-2 HRP DAB Broad-2 Detection system kit (GBI, Mukilteo, USA). Subsequently, these sections were counterstained with Harris’s hematoxylin (YD Diagnostics, Yongin, Korea).

We determined the MVD with CD34 immunostained tumor section. We counted the microvessels in the highest CD34 positive neovascularization areas (hot spots) of the tumor stroma. These were identified on the scan view in accordance with the method described by Weidner N (18). In the Notch1 immunostained slides, the highest Notch1 positive neovascularization areas (hot spots) were selected and counted Notch1 positive microvessels (Notch1 MVD). In the CD34 immunostained slides, the corresponding areas to Notch1 hot spots were selected and counted CD34 positive microvessels (CD34 MVD). The ratio of Notch1 MVD to CD34 MVD was defined as endothelial-Notch1 as previously described^31^. The EC Notch1 was classified into EC Notch1 low group or EC Notch1 high group based on the cutoff value. The cutoff value of EC Notch1 was set at the median expression value.

### Statistical analysis

For analysis of characteristics of young age breast cancer, independent t test or Mann-Whitney test was used for continuous variables, and Chi-square or Fisher’s exact test was used for categorical variables. The correlation between MR parameters and pathologic factors was analyzed using Pearson or spearman correlation coefficient. The association between recurrence and baseline and clinical characteristics were performed to logistic regression analysis. All statistical analyses were used by SAS Version 9.4 (SAS Institute Inc, Cary, NC) and p value below 0.05 was considered statistically significant.

## Supplementary Information

Supplementary Information 1.

Supplementary Information 2.
